# Adipocyte hyperplasia: the primary mechanism of supraspinatus intramuscular fat accumulation after a complete rotator cuff tendon tear: a study in the rabbit

**DOI:** 10.1080/21623945.2019.1609201

**Published:** 2019-04-29

**Authors:** Guy Trudel, Hans K. Uhthoff, Kayleigh Wong, Josée Dupuis, Odette Laneuville

**Affiliations:** a Bone and Joint Research Laboratory, Department of Medicine, Department of Biochemistry, Microbiology and Immunology, University of Ottawa, Ottawa, Ontario, Canada; b Department of Medicine, Faculty of Medicine, University of Ottawa, Ottawa, Ontario, Canada; c Department of Surgery, Faculty of Medicine, University of Ottawa, Ottawa, Ontario, Canada; d Department of Biostatistics, Boston University School of Public Health, Boston, MA, USA; e Department of Biology, University of Ottawa, Ottawa, Ontario, Canada

**Keywords:** Adipocytes, fat clumps, hyperplasia, hypertrophy

## Abstract

Intramuscular fat (IMF) accumulates in muscles of the rotator cuff after tendon tear. The number and cross-sectional area of fat clumps and of adipocytes were quantified on osmium tetroxide stained sections of the proximal, middle and distal quarters of SSP muscles 4, 8 and 12 weeks after SSP tendon division in a rabbit model. Linear mixed-effects models were fitted to the data and statistical significance was evaluated by ANOVA. Both the number (P<0.001) and cross-sectional area (P<0.0005) of fat clumps increased after tendon detachment while time had no significant effect (both at P>0.01). IMF accumulation was more important in the distal quarter of detached SSP muscle near tendon sectioning and characterized by increases of the number (P<0.0005) and cross-sectional area of fat clumps (P<0.0005) compared to the proximal quarter. Adipocyte number increased after tendon detachment (P<0.0005) and over time (P<0.01). The cross-sectional area of adipocytes increased in the detached group compared to controls (P<0.01) while time had no significant effect (P>0.01). Interestingly, the number of adipocytes in the distal quarter increased (P<0.0005) but the cross-sectional area was smaller (P<0.0005) compared to adipocytes in the proximal quarter. Adipocyte hyperplasia localized near tendon sectioning was the main contributor to fat accumulation in the detached SSP muscles.

## Introduction

The rotator cuff tear is the most common tendinopathy in humans and over 200,000 cuff repairs are performed annually in the United States [1,2]. The decreased morbidity associated with arthroscopic repairs has contributed to the popularity and broad indications for this surgical intervention [1,3]. Tendon reattachment even if biomechanically strong at the time of repair often fails and approximately 50% of patients with full-thickness tears of the rotator cuff report symptoms at 6 months after surgery [1,4,5].

**Figure 5.**
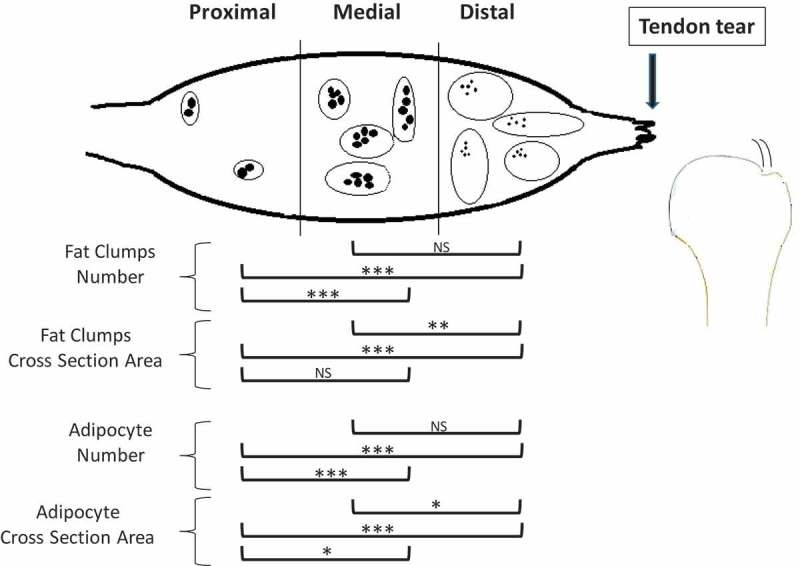
Schematic representation of the changes in the number and cross-section area of fat clumps and of adipocyte number in the proximal, medial and distal SSP muscle after a complete SSP tendon detachment. IMF increased closer to the tendon tear compared to the proximal SSP muscle. Detached muscles had more clumps in the distal and medial sections and of larger size in the distal section. There were more adipocytes in the distal and medial detached SSP muscles compared to proximal and cross-sectional area was smaller in the distal SSP muscle. The fat clumps are represented by ovals and adipocytes by smaller filed black shapes. Results from the statistical analysis are indicated: 0.001 ≤ P < 0.01 (*), 0.0005 ≤ P < 0.001 (**), P < 0.0005 (***).

The unsatisfactory success of rotator cuff repair surgeries has been attributed in many cases to muscle atrophy and fat accumulation both assessed by medical imaging methods [6–8]. The benefits of arthroscopy to repair the cuff and of advanced imaging methods to measure rotator cuff muscle fat content are undeniable but enhancing postoperative outcomes remain a challenge and basic knowledge on the mechanisms of intramuscular fat accumulation is needed [9–11].

Animal models of rotator cuff tendon injury and repair capture important aspects of the human disease [12–16]. Imaging of the rabbit’s SSP muscle documented fat accumulations both extra- and intra-muscular, and were evident as early as 4 weeks after SSP tendon detachment and progressed up to 12 weeks [17]. The fat signal increased from proximal-to-distal with the highest amount of fat detected in the distal quarter of the SSP muscle, the site closer to tendon detachment [17]. Both fat accumulation and muscle atrophy were present at week 1 and 2 after immediate repair but only fat accumulation persisted at 6 weeks [18,19]. In a different study, delayed tendon reattachment did not reverse SSP fat accumulation [20]. The rabbit experimental model of rotator cuff tear and repair reproduced accurately the human pathology and represents a valuable avenue to decipher the pathophysiology of IMF accumulation associated with rotator cuff tear [12,21].

The mechanisms for adipose tissue expansion have been studied extensively. In the context of obesity resulting from a high-fat diet, large fat accumulations are noticeable in subcutaneous and visceral deposits [22–24]. Overnutrition induced adipocyte hypertrophy in upper-body subcutaneous fat while a cycle between hypertrophy and hyperplasia characterized deposits below the waist [25]. The IMF deposit, considered a small fat deposit, is made up of white adipocytes and its accumulation characterizes late stages of muscular dystrophies [24,26]. The pathophysiology of adipocytes leading to IMF accumulation associated with rotator cuff tear remains unknown.

We hypothesized that IMF accumulation observed after rotator cuff tears results from adipocyte hypertrophy rather than hyperplasia leading to the enlargement of resident muscle fat clumps. The purpose of the current study was to characterize, at the microscopic level and over time, the expansion of the adipose tissue in the SSP muscle of rabbits after detachment of the distal SSP tendon.

## Materials and methods

### Animals and surgical procedure

This study was approved by the University of Ottawa Animal Care Committee. Adult female New Zealand rabbits (n = 45) weighing 3.0 kg were purchased from Charles River, Saint-Constant, Quebec, Canada and allowed to acclimate for one week upon arrival. For the experimental group, a supraspinatus tenotomy was performed unilaterally in 30 rabbits by sectioning completely the SSP tendon from the greater tuberosity of the humerus using a surgical blade under general anaesthesia [14]. Left and right shoulders were alternated. To prevent postoperative adhesions, the stump of the tendon was wrapped with a polyvinylidene membrane (5 µm, Durapore, Millipore, Bedford MA USA). Animals were housed individually, divided into three equal groups, killed at 4, 8 or 12 weeks after surgery and the operated shoulders were collected for histological analysis. For the control group, 15 unoperated rabbits were equally divided into three groups, killed at 4, 8 and 12 weeks and both shoulders were collected. The harvesting method of shoulders was described in our previous publication. Complete SSP muscles were dissected from the scapula, wrapped and frozen at −20°C until processed for histology analysis [17]. Radiology and macroscopic data on this group of animals have already been reported [17]; the current microscopy analysis at the cellular level builds on those studies.

### Histology specimen preparation

Harvested SSP muscles were fixed in 4% paraformaldehyde and rinsed twice for 1 h in phosphate buffered saline to begin processing for histology. Muscle specimens were frozen to preserve fat structures during sectioning. From each muscle, three cross-section slices of 1-mm thickness were cut at the proximal quarter, middle-half, and distal quarter sites of the supraspinatus muscle. Muscle slices were stained for 2 weeks with 5% potassium dichromate and 2% osmium tetroxide followed by paraffin embedding [14]. Using a microtome, 6µm-thick microscopy slides were prepared. Fixation in osmium tetroxide stained adipocytes black.

### Histology evaluation and microscopy image analysis

A total of 180 slides from detached tendons and from unoperated tendons, at time points 4, 8 or 12 weeks, in the proximal, middle or distal quarters of the SSP muscle were analysed by light microscopy (Table 1).

**Table 1.** Summary of the samples studied including numbers of rabbits, shoulders, and tissue sections for both fat clump and adipocyte analyses.

SSP Muscle Quarter/Weeks	Detached vs Control	Rabbits (N)	Shoulders (N)	Muscle Sections (Clumps) (N)	Muscle Sections (Cells) (N)
Proximal Quarter					
4	Detached	10	10	8	10
	Control	5	10	10	10
8	Detached	10	10	10	10
	Control	5	10	10	10
12	Detached	10	10	9	10
	Control	5	10	10	10
Middle Quarter					
4	Detached	10	10	10	10
	Control	5	10	9	10
8	Detached	10	10	8	10
	Control	5	10	10	10
12	Detached	10	10	9	10
	Control	5	10	10	10
Distal Quarter					
4	Detached	10	10	10	10
	Control	5	10	10	10
8	Detached	10	10	10	10
	Control	5	10	10	10
12	Detached	10	10	10	10
	Control	5	10	10	10
				**Fat Clumps**	**Adipocytes**
Total Muscle Sections/Fields Analyzed	Detached			84	90 (270 fields)
	Control			89	90 (270 fields)
Total Fat Clumps/Adipocytes Analyzed	Detached			18,542	10,389
	Control			14,345	6706

**Figure 1.**
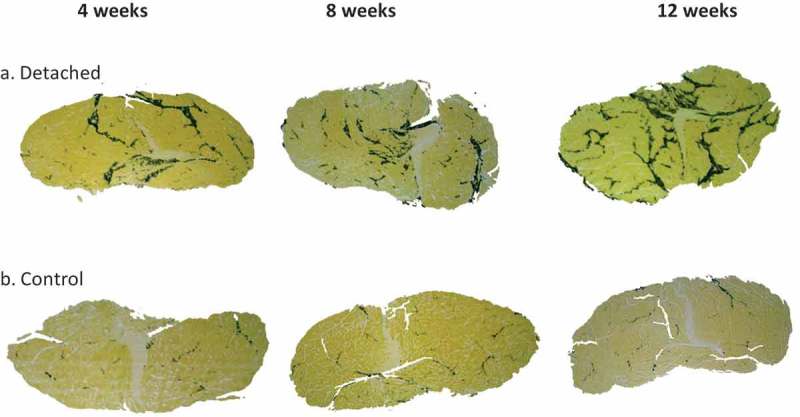
Representative micrographs of IMF accumulation in the distal quarter of the SSP muscle cross-sections. (a). SSP muscle sections at 4, 8 and 12 weeks after tendon detachment. (b). SSP muscle sections in control animals at the same time points. IMF was stained using osmium tetroxide and is visible at black-stained areas. Note the higher accumulation of fat in the tendon detached group compared to controls at all time points studied. Original magnification at 6.7x.

Fat clumps were measured on entire SSP muscle cross-sections digitized at 6.7x magnification and backgrounds were cropped using Corel Photo-Paint 11. Images were then imported for computer-assisted quantitative image analysis using software ImageJ software (version 1.34s; National Institute of Health, Bethesda, MD, USA). Scales were set by using calliper measurements of two reference points on the slide and converted to pixels. Pictures were converted into binary black and white images (8-bit; grey scale). A fat clump was defined as an area of fat stained black, not in contact with another stained area (Figure 1). The ‘threshold’ function was manually adjusted to select only black pixels. The ‘watershed’ function was used to mark the boundaries of individual fat clumps. The ‘analyse particle’ command was used to measure clump numbers and areas with ‘cellularity’ set at 0–1 and ‘size’ set at 0-infinity. The command ‘measure all’ was used to automatically generate all measurements.

**Figure 2.**
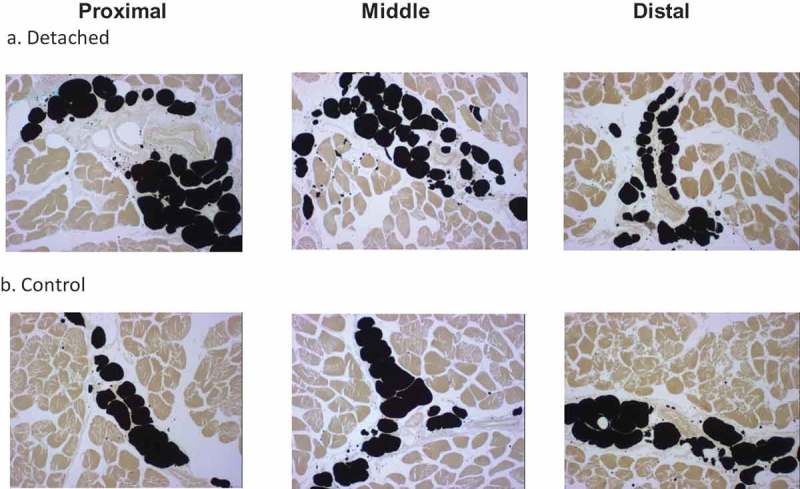
Representative micrographs of adipocytes in the proximal, middle and distal quarters of the SSP muscle. (a). SSP muscle sections at 12 weeks after tendon detachment. (b). SSP muscle sections from control age-matched animals. Adipocyte vacuoles stained black using the osmium tetroxide protocol described in the Method section. Note the increased number of smaller adipocytes in the tendon detached group compared to controls. Original magnification at 25x.

Adipocyte number per field and average cross-sectional area were measured using computer-assisted image analysis of the same microscopic slices captured at 25x magnification. Three different fields of equal and fixed areas (0.149 mm^2^ each) were chosen using the following criteria: included black staining, not contiguous with the other selected fields, included minimal empty space, and included at least one blood vessel. No field overlapped. The three different fields analysed in each 3 muscle sections (proximal, middle and distal) in 10 rabbits per time point (4, 8 and 12 weeks) group in each of detached SSP and control groups amounted to a total of 540 fields. Representative images from distal quarters at 4, 8 and 12 weeks after tenotomy and corresponding controls are presented in Figure 2. To measure adipocyte number and size, we again used ImageJ, images were converted to 8-bit grey-scale pictures. Default settings of the ‘thresholding’ function were used to select only the black-stained adipocytes. Applying the threshold converts the image to black and white, displaying only adipocytes. The ‘watershed’ function was used to separate individual cells, and ‘analyse particles’ was used to count and measure the cross-section area of adipocytes. Minimum size was set at 350 pixels (to remove artefacts originating from microtomy) and circularity of 0.5 (to remove any cells cut off at the edges of the picture). ImageJ was calibrated by using a scale bar to convert pixels into mm^2^.

### Data and statistical analysis

**Figure 3.**
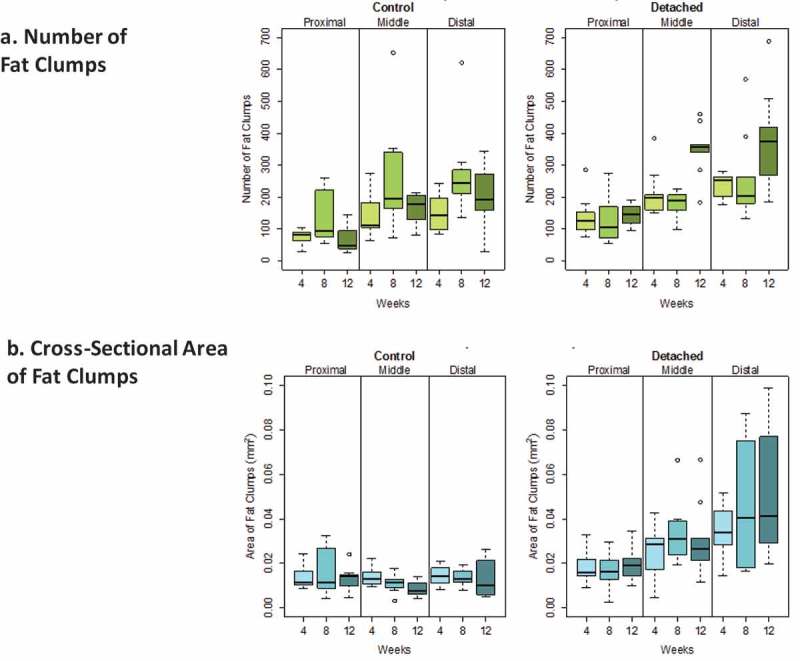
Boxplots showing the distribution of intramuscular fat clump numbers (a) and cross-sectional area (b) (mm^2^) for SSP tendons detached for 4, 8 and 12 weeks and for age-matched controls. Horizontal lines in the boxes represent the median values, limits of the boxes represent upper and lower quartiles, lines extending vertically from boxes represent variability outside the boxes and outliers are plotted as individual points. The dispersion of the number of fat clumps was similar for both detached and controls. A large variability in the fat clump cross-sectional areas was observed for the detached group in the distal quarter at 8 and 12 weeks after detachment and displayed in the large sizes of the boxes for these two groups compared to controls.

**Figure 4.**
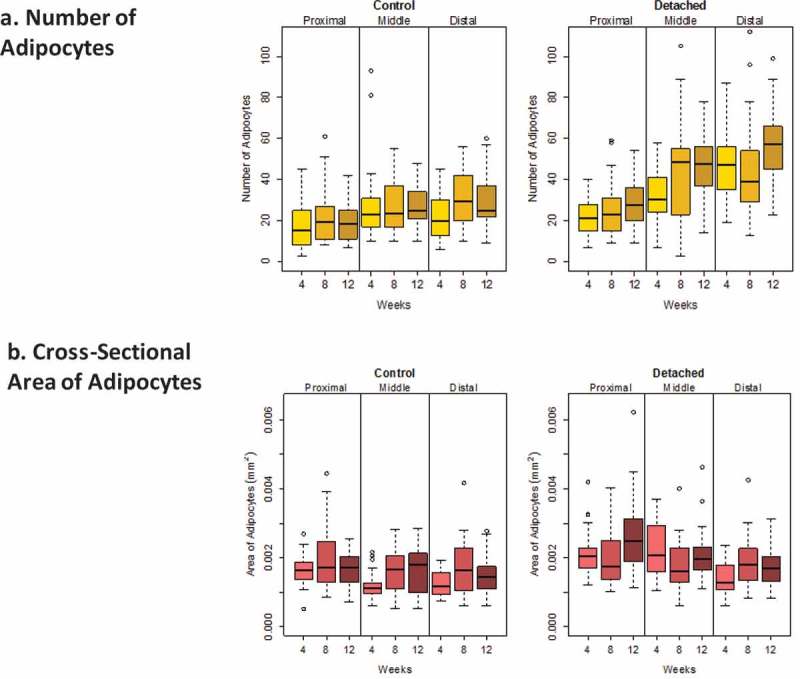
Boxplots showing the distribution of intramuscular adipocyte numbers (a) and cross-sectional area (b) (mm^2^) for SSP tendons detached for 4, 8 and 12 weeks and for age-matched controls. Horizontal lines in the boxes represent the median values, limits of the boxes represent upper and lower quartiles, lines extending vertically from boxes represent variability outside the boxes and outliers are plotted as individual points. The dispersion in the number of adipocytes was larger in the middle and distal quarters of the SSP muscle in the detached group compared to controls. The dispersion in adipocyte cross-section area was comparable in both groups.

Descriptive statistics displayed the medians and interquartile ranges of the four outcomes measured; the number and cross-sectional area of both fat clumps and adipocytes. We first explored the distribution of the fat clumps and adipocytes outcomes because a skewed distribution of adipocytes diameter had previously been described [27]. Our data for fat clumps and for adipocytes confirmed an asymmetrical distribution (Figures 3 and 4); median lines were off centre of the interquartile boxplots and upper and lower whiskers for the same box were of different size indicative of a skewed distribution. Skewed data distribution was log transformed to meet the normality assumptions for ANOVA and regression-based statistical analyses. In this paper, non-log-transformed data were reported as descriptive statistics whereas log-transformed data were used in the quantitative statistical analyses. Linear mixed-effects (LME) models were fitted to the data, and statistical significance for the four outcomes was evaluated by ANOVA considering the different muscle locations as a single fixed-effect factor and similarly for the three time points after detachment, but using a random effect to account for correlation between measurements taken from the same rabbit. Post hoc analysis was conducted when significant differences were observed also using LME models, and pairwise comparisons of the fixed effects were performed. The fixed effects were introduced as a single term in the equations and considered without interaction. For each of the four outcomes evaluated; fat clumps number, fat clumps cross-sectional area, adipocyte number and adipocytes cross-section area, the following equation was applied: Outcome ~ log(μ) + β_1_ time + β_2_ location + β_3_ detachment + error (1|rabbit).

LME modelling also accounted for two characteristics of our study design with potential influence on the outcomes of the statistical analyses [28]. First, the fat clumps and adipocytes were measured in three quarters of the same SSP muscle and are not independent observations. Second, number and cross-sectional area outcomes are potentially influenced by a random effect corresponding to animals and by fixed effects including SSP tendon detachment, quarter of the muscle, and time after SSP tendon detachment. P-values for the calculated coefficient of individual fixed effect estimates were used to determine their contribution to fat accumulation. Significance was determined according to P values at: 0.001 ≤ P < 0.01 (*), 0.0005 ≤ P < 0.001 (**), P < 0.0005 (***). We considered p < 0.01 to be statistically significant because of the multiple outcomes and models analysed. All descriptive and statistical analyses were performed using the open-source programming environment R [29] and the lmerTest package [30].

## Results

Table 1 describes the samples analysed including numbers of rabbits, shoulders, tissue sections and fields in detached and control SSP muscles. Seven out of 360 slides showed poor staining quality in some areas and were omitted from the low magnification microscopy analysis (fat clumps) (Table 1). The total number of stained fat clumps was 18,542 for the detached groups and 14,345 for the control groups. The total number of adipocytes analysed was 10,389 for the detached group and 6706 for the control group. Representative micrographs of osmium tetroxide stained SSP muscle sections and of fat clumps and adipocytes are presented in Figures 1 and 2.

### Descriptive statistics of fat clump numbers and areas

The average number (± standard error) of fat clumps for all quarters of all the detached SSP muscles was 223.1 ± 87.5 and for all the control muscles 160.8 ± 70.5. Average fat clump areas were 0.031 ± 0.011 mm^2^ for detached SSP muscles and 0.013 ± 0.023 mm^2^ for controls (Figure 3).

### Quantitative analysis of fat clumps after SSP tendon detachment

SSP tendon detachment was associated with increased fat clump numbers (P < 0.001) and area (P < 0.0005) in SSP muscles compared to controls (Table 2). Time after SSP tendon detachment did not significantly influence fat clump numbers and area (both at P > 0.01). The muscle location (distal, middle or proximal) was strongly associated with increases of fat clumps number (P < 0.0005) and area (P < 0.0005) (Table 2). There were significantly more fat clumps in the distal quarter compared to the proximal quarter (P < 0.0005) but not significantly different from the medial quarter (P > 0.01). The proximal quarter contained fewer fat clumps compared to the medial quarter (P < 0.0005). Fat clumps were significantly larger in the distal quarter compared to the medial (P < 0.001) and proximal quarters (P < 0.0005) while proximal and medial quarters were not significantly different (P > 0.01) (Table 2).

**Table 2.** Summary of ANOVA and of the post hoc linear mixed-effects model for the fat clump number and cross-section area. 0.001 ≤ P < 0.01 (*), 0.0005 ≤ P < 0.001 (**), P < 0.0005 (***).

Variable	ANOVA (P Value)		
Detachment	P < 0.001 (**)		
Week	P > 0.01		
Location	P < 0.0005 (***)		
	**Fat clump number ~ log(μ) + β_1_ time + β_2_ location + β_3_ detachment + error (1|rabbit)**		
	**β coefficient**	**Std Error**	**P Value**
Distal vs Medial	−0.115	0.077	P > 0.01
Distal vs Proximal	−0.787	0.076	P < 0.0005 (***)
Proximal vs Medial	0.672	0.076	P < 0.0005 (***)
Fat Clump Cross Section Area			
Variable	**ANOVA (P Value)**		
Detachment	P < 0.0005 (***)		
Week	P > 0.01		
Location	P < 0.0005 (***)		
	**Fat clump area ~ log(μ) + β_1_ time + β_2_ location + β_3_ detachment + error (1|rabbit)**		
	**β coefficient**	**Std Error**	**P Value**
Distal vs Medial	−0.287	0.084	P < 0.001 (**)
Distal vs Proximal	−0.399	0.082	P < 0.0005 (***)
Proximal vs Medial	0.111	0.083	P > 0.01

### Descriptive statistics of adipocyte numbers and areas

Detached SSP muscles had on average 38.5 ± 11.7 adipocytes per field of view compared to 24.8 ± 4.8 for controls. Average adipocyte cross-sectional area was 0.0020 ± 0.0003 mm^2^ for detached SSP muscles compared to 0.0016 ± 0.0003 mm^2^ for controls (Figure 4).

### Quantitative analysis of adipocytes after SSP tendon detachment

SSP tendon detachment was associated with increased adipocyte numbers (P < 0.0005) and cross-section area (P < 0.01) in SSP muscles compared to controls (Table 3). Time after SSP tendon detachment significantly increased adipocytes number (P < 0.01) but had no significant influence on adipocytes cross-section area (P > 0.01). Detached SSP muscles had significantly more adipocytes at week 12 (P < 0.01) compared to week 4 (Figure 4). The number of adipocytes was not significantly different between week 4 and week 8 (P > 0.01) and between week 8 and week 12 (P > 0.01). Muscle location (distal, middle or proximal) was associated with increased adipocyte numbers (P < 0.0005) and cross-section areas (P < 0.0005) (Table 3). There were significantly more adipocytes in the distal quarter (P < 0.0005) compared to the proximal quarter but not compared to the medial quarter (P > 0.01). The medial quarter contained more adipocytes than the proximal quarter (P < 0.0005). Adipocytes were significantly smaller in the distal quarter (P < 0.01) compared to the medial and to the proximal quarters (P < 0.0005) (Table 3 and Figure 4). Adipocyte in the medial quarter was also smaller than in the proximal quarter (P < 0.01).

**Table 3.** Summary of ANOVA and of the post hoc linear mixed-effects model of adipocyte number and cross-section area. 0.001 ≤ P < 0.01 (*), 0.0005 ≤ P < 0.001 (**), P < 0.0005 (***).

Variable	ANOVA (P Value)		
Detachment	P < 0.0005 (***)		
Week	P < 0.01 (*)		
Location	P < 0.0005 (***)		
	**Adipocyte number ~ log(μ) + β_1_ time + β_2_ location + β_3_ detachment + error (1|rabbit)**		
	**β coefficient**	**Std Error**	**P Value**
Week 4 vs 8	0.147	0.072	P > 0.01
Week 4 vs 12	0.239	0.072	P < 0.01 (*)
Week 12 vs 8	−0.091	0.072	P > 0.01
Distal vs Medial	−0.09	0.057	P > 0.01
Distal vs Proximal	−0.493	0.057	P < 0.0005 (***)
Proximal vs Medial	0.403	0.057	P < 0.0005 (***)
Adipocyte Cross Section Area			
Variable	**ANOVA (P Value)**		
Detachment	P < 0.01 (*)		
Week	P > 0.01		
Location	P < 0.0005 (***)		
	**Adipocyte area ~ log(μ) + β_1_ time + β_2_ location + β_3_ detachment + error (1|rabbit)**		
	**β coefficient**	**Std Error**	**P Value**
Distal vs Medial	0.117	0.041	P < 0.01 (*)
Distal vs Proximal	0.256	0.041	P < 0.0005 (***)
Proximal vs Medial	−0.138	0.041	P < 0.01 (*)

## Discussion

We characterized intramuscular fat accumulation in the SSP muscle in the rabbit model of rotator cuff tear. SSP tendon detachment produced an increase number of larger fat clumps and an increased number of smaller adipocytes in the distal quarter of the SSP muscle near the site of tendon tear (Figure 5). Time after tendon detachment significantly increased the number of adipocytes. Our hypothesis based on literature on obesity that: IMF accumulation after rotator cuff tears resulted from adipocyte hypertrophy rather than hyperplasia was infirmed. The current study established that adipocyte hyperplasia was the main contributor to fat clump enlargements and explained SSP IMF expansion up to 12 weeks after tendon detachment.

Fat tissue has been described to expand via two mechanisms; adipocyte hyperplasia (the increase of the number of adipocytes) and adipocyte hypertrophy (the increase in individual adipocyte size) [24,31]. Knowledge of adipocytes’ behaviour originates mostly from obesity research and expansion of sub-cutaneous white fat deposits. Experiments from the 1970s showed that overfeeding combined with reduced energy expenditure over several months resulted in an important increase of adipocyte size without significant changes in the number adipocytes [32]. Consistently, the turnover of human subcutaneous adipocytes each year is very low, approximately 8%, resulting in little change in adipocyte number and emphasizing the importance of adipocyte hypertrophy in the expansion of fat tissue in the context of obesity [33]. There is evidence for regional differences in adipocytes behaviour in human obesity. While adipocyte hypertrophy characterizes upper body sub-cutaneous fat, adipocytes cycling between hyperplasia and hypertrophy characterized deposits below the waist as obesity progresses upon high-fat feeding [23,25,34]. Our results indicate that expansion of IMF in the SSP muscle present significant similarities with subcutaneous fat deposits located below the waist; fat expansion resulted from adipocyte hyperplasia at least within the first 12 weeks after tendon detachment.

Intramuscular adipocytes in the current study were approximately 0.002 mm^2^ or 25 microns in diameter (assuming circular shape of adipocytes, πr^2^). This was smaller than mature white adipocytes with approximately 110 microns in diameter (ranges from 20 to 300 microns) [27,35]. Smaller adipocytes less than 10 microns in diameter were previously described in rat epididymal fat deposit [27,36]. Considering the published spectrum of sizes for white adipocytes, intramuscular adipocytes were therefore characterized as small in both healthy and detached SSP muscles.

Increased adipocyte cellularity is indicative of the mechanism of adipogenesis taking place in the detached SSP muscle. The observations of increased adipocyte number in combination with small cross-section areas in the distal quarter where fat accumulation was the most important suggested the presence of newly formed cells. Pre-adipocytes are smaller in size than mature adipocytes [37–39]. Newer adipocytes of smaller size driving the average adipocyte size lower are a potential explanation of the lower adipocyte size in the distal SSP muscle. Moreover, the persistence of smaller average size adipocytes 12 weeks after tendon detachment suggests that, rather than maturing and growing to reach proximal size, new adipocytes that were present 4 weeks after detachment have remained small or new adipocytes were continuously generated in the distal detached SSP muscle.

The identity of the precursor cells contributing to the increased intramuscular adipocyte hyperplasia is actively investigated. Adipocytes derive from pre-adipocytes which themselves differentiate from mesenchymal precursor cells [39]. Adipocytes can also originate from existing mesenchymal tissue in the muscle [37]. Four candidate muscle cells able to generate adipocytes have been described; a population of fibrocyte/adipocyte progenitors, muscle satellite cells, pericytes [35] and bone marrow-derived cells [40]. During skeletal muscle degeneration, adipocytes were demonstrated to derive from a population of bipotent progenitors residing within muscles and different from muscle progenitors [38,40]. There is experimental support in mice for these cells as the source of SSP muscle adipocytes after rotator cuff tear [41,42]. Satellite cells are also a population of primary cells residing in muscles with the ability to differentiate into adipocytes *in vitro*. [43–45] Fibrocyte/adipocyte progenitor and satellite cells may be activated locally in the distal quarter of the SSP muscle to produce adipocytes. The trigger may be the absence of forces transmitted to the muscle through the intramuscular tendon fibres after tendon detachment. Altered mechanical activity at the myotendinous junction may also explain the more prominent fatty accumulation at the distal quarter of the SSP muscle[46]. Pericytes physically associated with the walls of intra-adipose blood vessels showed the potential to differentiate into adipocytes *in vitro* [47]. Interestingly, the habitual presence of blood vessels in the vicinity of the fat cells was used as criteria to select the fields for measurements. But the vascularization of skeletal muscles enters through the middle half of the SSP muscle and is distributed to the distal and proximal portions [48]. The muscle vascular distribution is inconsistent with the IMF we observed. The identity of the precursor cell(s) differentiating into adipocytes is only speculative at this time and all four previously identified precursors are potential candidates.

The direct clinical implication of the current findings of adipocyte hyperplasia as the mechanism for fatty accumulation lies in its treatment. Successful treatment of rotator cuff tear and IMF accumulation will require a strategy to reduce the number of adipocytes. This is a significant challenge since extensive literature indicates that adipocyte hypertrophy in obesity can be combatted by reducing caloric intake and increasing energy expenditure [22–24]. However, this approach is unsuccessful for adipocyte hyperplasia. Intramuscular white fat, similar to other white fat deposits is characterized by a persisting number of adipocytes. Once adipocyte number increases, they are durable and difficult to lose [24]; important weight loss resulted from a reduction in adipocyte volume but not overall number [25,32]. This concept is consistent with the literature on reversibility of fat accumulation after rotator cuff tear. While initially believed to recover after successful tendon repair, numerous experimental as well as clinical studies have shown that fat accumulation is largely irreversible. Uhthoff et al. [19] showed that animals with reattachment immediately after tear could recover muscle volume but did not reverse fat accumulation. Delayed repair also failed to reverse fat accumulation [18–21]. These four studies used precise, invasive as well as radiologic measures and followed up SSP muscles for 3 months after repair. Clinically, 38 patients showed no reversal of fatty accumulation 12–15 months postoperatively [47,49], 35 patients showed progression of fat accumulation 6 months after repair[23], and 47 patients followed between 60 and 133 months also showed progression of the fatty content of the rotator cuff muscles [50,51]. The lack of reversibility of IMF accumulation may indicate a need for a fast intervention at repairing SSP tendon tears.

Limitations of the current study include: 1) the anatomy of the rabbit rotator cuff muscles differs from human; 2) sectioned tendons were wrapped in polyvinylidine fluoride membranes to prevent the formation of adhesions and this is not the case in humans; 3) changes were studied during the first 12 weeks after tendon detachment; in clinical practice, longer delays before surgical repair of the SSP tendon tear are common; 4) tendon sectioning performed to achieve complete detachment is different than tendon tear; 5) some fat deposits in human have no precise correlates in animals and vice versa; 6) the osmium fixation method of determining adipocyte size and numbers is only possible in experimental studies. In spite of those limitations, the rabbit model of rotator cuff tear has stood out in its potential to replicate the clinical findings of fat accumulation.

## Conclusion

This study established adipocyte hyperplasia and increased fat clump numbers and size as the main mechanism causing fat accumulation in the SSP muscle with 12 weeks after a rotator cuff tear. The changes were predominant in the distal quarter of the SSP muscle, near the tendon tear where adipocyte number but not size increased. The trigger for adipocyte hyperplasia and the cell precursor(s) remains to be identified as a next step in the search for better SSP repair outcome.
